# Methylation-Mediated Silencing of *RBP7* Promotes Breast Cancer Progression through PPAR and PI3K/AKT Pathway

**DOI:** 10.1155/2022/9039110

**Published:** 2022-10-13

**Authors:** Hong Lin, Qizheng Han, Junhao Wang, Zhaoqian Zhong, Haihua Luo, Yibin Hao, Yong Jiang

**Affiliations:** ^1^The fifth Clinical Medical College of Henan University of Chinese Medicine, Henan University of Chinese Medicine, No. 33 Huanghe Road, Zhengzhou, 410105 Henan, China; ^2^Guangdong Provincial Key Laboratory of Proteomics, State Key Laboratory of Organ Failure Research, Department of Pathophysiology, School of Basic Medical Sciences, Southern Medical University, No. 1023, South Shatai Road, Baiyun District, Guangzhou, 510515 Guangdong, China

## Abstract

Retinoid-binding protein7 (*RBP7*) is a member of the cellular retinol-binding protein (CRBP) family, which is involved in the pathogenesis of breast cancer. The study aims to illustrate the prognostic value and the potential regulatory mechanisms of *RBP7* expression in breast cancer. Bioinformatics analysis with the TCGA and CPTAC databases revealed that the mRNA and protein expression levels of *RBP7* in normal were higher compared to breast cancer tissues. Survival analysis displayed that the lower expression of *RBP7,* the worse the prognosis in ER-positive (ER^+^) breast cancer patients. Genomic analysis showed that low expression of *RBP7* correlates with its promoter hypermethylation in breast cancer. Functional enrichment analysis demonstrated that downregulation of *RBP7* expression may exert its biological influence on breast cancer through the PPAR pathway and the PI3K/AKT pathway. In summary, we identified *RBP7* as a novel biomarker that is helpful for the prognosis of ER^+^ breast cancer patients. Promoter methylation of *RBP7* is involved in its gene silencing in breast cancer, thus regulating the occurrence and development of ER^+^ breast cancer through the PPAR and PI3K/AKT pathways.

## 1. Introduction

Breast cancer is the most common cancer worldwide, accounting for 30% of female cancers [1]. Estrogen receptor-positive (ER^+^) breast cancer is driven by ER-mediated transcriptional activity, composing the major subtype (approximately 75%) of breast cancer [2]. Although endocrine therapy, including estrogen suppression and direct ER targeting, is widely applied in the treatment of ER^+^ breast cancer, acquired resistance often occurs and remains a major challenge for the treatment of ER^+^ patients [3]; thus, novel targets and effective therapeutic strategies for breast cancer patients are urgently needed.

Previous studies have confirmed that cellular retinol-binding protein (CRBP) family members play an important role in the pathological progression of breast cancer. CRBPs belong to the family of fatty acid-binding proteins and are required for vitamin A stability and metabolism [4]. Epigenetic silencing of CRBPs is a common event in cancers [5]. For example, Kuppumbatti et al. reported that CRBPs were underexpressed in 24% of human breast cancer [6]. Previous studies demonstrated that inhibition of the PI3K/AKT pathway by CRBPs was involved in the growth inhibition of mammary epithelial cells [7]. In breast epithelial cells, CRBP1 inhibits PI3K/AKT signalling through a retinoic acid receptor-dependent mechanism that regulates p85-p110 heterodimerization [8]. In addition, physiological retinoic acid receptor (RAR) activation is dependent on CRBP1-mediated retinol storage, and CRBP1 downregulation chronically compromises RAR activity, leading to loss of cell differentiation and tumor progression [9]. Thus, CRBPs may be used as a potential biomarker for the diagnosis and treatment of breast cancer.

Retinoid-binding protein 7 (*RBP7*), also named CRBP4, belongs to a clearly distinct CRBP subfamily, representing a relatively different mode of retinol binding for this protein. The *RBP7* gene is located on human chromosome 1p36.22, encoding a protein of 134 amino acids in length. The size of the translated exon sequences and intron position of *RBP7* is highly conserved. With a structure similar to other CRBPs, the *RBP7*-encoded protein binds all-trans-retinol with a lower binding affinity than other CRBPs [10]. It was reported that *RBP7* regulates the occurrence and development of many diseases. For example, the PPAR*γ*-*RBP7*-adiponectin pathway plays a protective role in hypertensive diseases by regulating transcriptional activity [11]. *RBP7* also plays an important role in adipose tissue during adipogenesis, cold exposure, and nutritional treatment [12]. Recent studies have demonstrated that *RBP7* is a strong prognostic biomarker contributing to the malignant phenotype in colon cancer [13]. However, as a novel member of the CRBP family, the clinical and prognostic significance of *RBP7* in breast cancer is still unknown, and its functional role in breast cancer has never been documented.

In this study, we first analyzed the differential expression of *RBP7* in breast cancer and normal tissues and evaluated the prognostic value of *RBP7* by using data from the TCGA and GEO databases. Next, we determined the localization of *RBP7* expression in breast cancer, the functional enrichment of its coexpressed genes, and the association between the mRNA expression and DNA methylation of *RBP7*. Then, we explored the association between *RBP7* and multiple molecular subtypes of breast cancer and the significant KEGG pathways involved in *RBP7* in ER^+^ breast cancer. Finally, we screened *RBP7*-targeting drugs from computational analysis of resistance (CARE) databases, which may provide new ideas for the treatment of breast cancer.

## 2. Materials and Methods

### 2.1. Gene Expression and Survival Analysis

The Human Protein Atlas [14, 15] (HPA; http://www.proteinatlas.org) database was used to illustrate *RBP7* mRNA distribution, protein expression, and immunohistochemical maps of *RBP7* in normal and breast cancer tissues. *RBP7* gene expression levels in pan cancers were identified in Tumor Immune Estimation Resource [16] (TIMER; https://cistrome.shinyapps.io/timer/) and ONCOMINE [17] (http://www.oncomine.org). UALCAN [18] (http://ualcan.path.uab.edu/index.html) and the Gene Expression Omnibus [19] (GEO; https://www.ncbi.nlm.nih.gov/geo/) were used to investigate the different expression of *RBP7* in normal and breast cancer tissues. To explore the prognostic role of *RBP7* expression in breast cancer, Gene Expression Profiling Interactive Analysis [20] (GEPIA; gepia2.cancer-pku.cn/#index), Kaplan–Meier plotter [21] (http://kmplot.com/), and PROGgeneV2 [22] (http://www.compbio.iupui.edu/proggene) were used to determine the prognostic significance. In this study, we analyzed the prognosis of *RBP7* in breast cancer with detailed hazard ratios (HRs) by setting the expression threshold at the medium or best cut-off and *a* log-rank *pvalue* less than 0.05.

### 2.2. Tumor Immune Single-Cell Hub (TISCH)

TISCH [23] (http://tisch.comp-genomics.org) integrates single-cell transcriptome profiles of nearly 2 million cells for 27 cancer types. In this study, we utilized the “multiple-dataset comparison” model to visualize the averaged gene expression distributed in single cells and the “Gene” module to display the heat map of the cell-type averaged expression of *RBP7*.

### 2.3. LinkedOmics

The coexpressed genes of *RBP7* were screened from the TCGA BRCA (breast invasive carcinoma) cohort through the “LinkFinder” module in LinkedOmics [24] (http://www.linkedomics.org/login.php) databases, and the correlative significance was tested by the spearman correlation coefficient. The top 50 positively and negatively correlated genes are presented as heat maps. Gene Ontology biological process (GO_BP) and Kyoto Encyclopedia of Genes and Genomes (KEGG) pathway analyses were performed with gene set enrichment analysis (GSEA) in the “LinkInterpreter” module.

### 2.4. *RBP7* DNA Methylation Analysis

Heat maps of *RBP7* in the cohort of breast cancer patients were constructed through data mining in TCGA BRCA by using the University of California Santa Cruz (UCSC) Xena [25, 26] (http://xena.ucsc.edu/). MethSurv (http://biit.cs.ut.ee/methsurv/) is used to survival analysis ground on CpG methylation patterns [27], we verified the methylation levels of probes of *RBP7,* and four of them with high methylation in the promoter were chosen to display the distribution of methylation under different clinical stages. SMART [28, 29] (http://www.bioinfo-zs.com/smartapp/) is to further identify the association of the mRNA expression and methylation of *RBP7*.

### 2.5. Bc-GenExMiner Online Tool and OSbrca

Based on common clinical parameters, we utilized Bc-GenExMiner (v4.7) [30] (http://bcgenex.ico.unicancer.fr) to analyze the expression data and survival curves of *RBP7* in different molecular subtypes of breast cancer, including ER, PR, and HER-2 (IHC). The OSbrca [31] (http://bioinfo.henu.edu.cn/BRCA/BRCAList.jsp) was utilized to validate prognostic value of *RBP7* in breast cancer.

### 2.6. Protein–Protein Interaction (PPI) Network Analysis

PPI network analysis of *RBP7* was conducted in the STRING (https://string-db.org/) database [32]. The regulatory relationships between genes were visualized *via* Cytoscape (ver. 3.4.0). Then, we use the starBase v3.0 [33] (http://starbase.sysu.edu.cn/index.php) to analyze the correlation between *RBP7* and PIK3R3 in breast cancer.

### 2.7. Differentially Expressed Genes (DEGs) Analysis

We used the Limma package to screen DEGs by filtering the *p*.*adj* value of Student's *t*-test and the fold change (FC) and dividing the DEGs into two groups with high or low *RBP7* expression. A volcano plot was generated by using the ggplot2 R software package to display the DEGs with statistical significance, i.e., *p*.adjust value <0.05 and absolute FC value >1. KEGG pathway analysis was performed on those DEGs by using the cluster profiler package, and the pathways with statistical significance (adjusted *p* < 0.05) were visualized by hierarchical clustering of a heat map [34].

### 2.8. Computational Analysis of Resistance (CARE)

A positive CARE score represented a high expression value, which was related to drug response in CARE [35] (http://care.dfci.harvard.edu/), and vice versa. In this study, we utilized 3 databases, i.e., Cancer Cell Line Encyclopedia (CCLE), Cancer Therapeutics Response Portal (CGP), and Genomics of Drug Sensitivity in Cancer (CTRP), to analyze the drugs targeting *RBP7*.

### 2.9. SwissDock

The PDF file of the *RBP7* protein was downloaded from the RCSB Protein Data Bank (PDB) database, and the ligand and water molecules were then removed by using PyMOL software. The mol2 file of the nilotinib small molecule was downloaded from the PubChem database and converted using Open Babel software. Finally, we uploaded the two files to the SwissDock [36] (http://www.swissdock.ch) page for docking.

## 3. Results

### 3.1. Gene Expression Profiles of *RBP7* in Normal and Cancer Tissues

**Figure 1:**
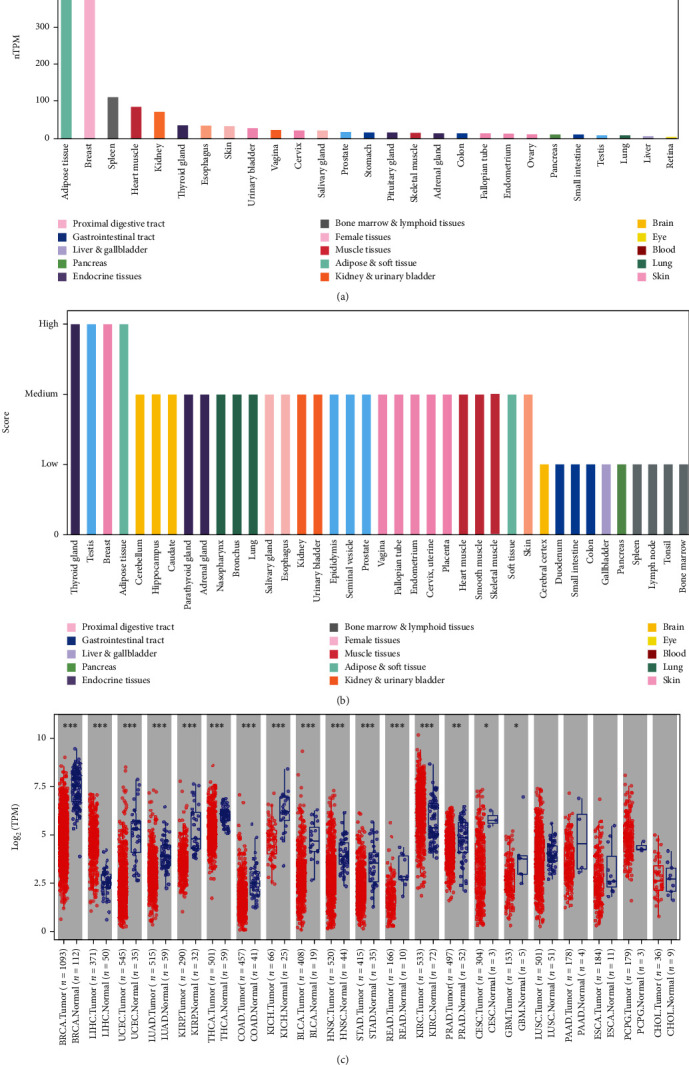
The expression of *RBP7* in different human tissues and pan cancers. (a) *RBP7* expression profiles in normal human tissues from the GTEx project. nTPM, normalized protein-coding transcripts per million. (b) The protein expression of *RBP7* in normal human tissues. (c) The mRNA expression levels of *RBP7* in different cancer types were explored by TIMER. It is presented by ranking the *p* value. ^∗^*p* < 0.05, ^∗∗^*p* < 0.01, ^∗∗∗^*p* < 0.001.

We utilized the HPA database to analyze the mRNA and protein expression profiles of *RBP7* in human normal tissues. We found that the *RBP7* mRNA expression was mainly in breast and adipose tissues in normal human tissues by using the GTEx (genotype-tissue expression) database (Figure 1(a)). Consistently, the protein expression of *RBP7* was highly expressed in the breast and adipose tissues (Figure 1(b)). The mRNA expression levels of *RBP7* were explored by TIMER in many cancer types. Additionally, the results revealed that *RBP7* mRNA expression levels were significantly lower in most cancer samples than their corresponding normal samples, including breast invasive carcinoma (BRCA), uterine corpus endometrial carcinoma (UCEC), and lung adenocarcinoma (LUAD). Besides, the data also showed that *RBP7* expression was aberrantly higher in liver hepatocellular carcinoma (LIHC) and kidney renal clear cell carcinoma (KIRC) (Figure 1(c)).

To further verify the significance of *RBP7* expression in cancers, the differential expression of *RBP7* in tumor and normal tissues was analyzed by using ONCOMINE. We found that *RBP7* was overexpressed in liver cancer and lymphoma, but decreased expression of *RBP7* was found in brain and CNS cancer, breast cancer, esophageal cancer, head and neck cancer, leukemia, and ovarian cancer (Supplementary Figure 1A).

### 3.2. Prognostic Value of *RBP7* Expression in Breast Cancer

**Figure 2:**
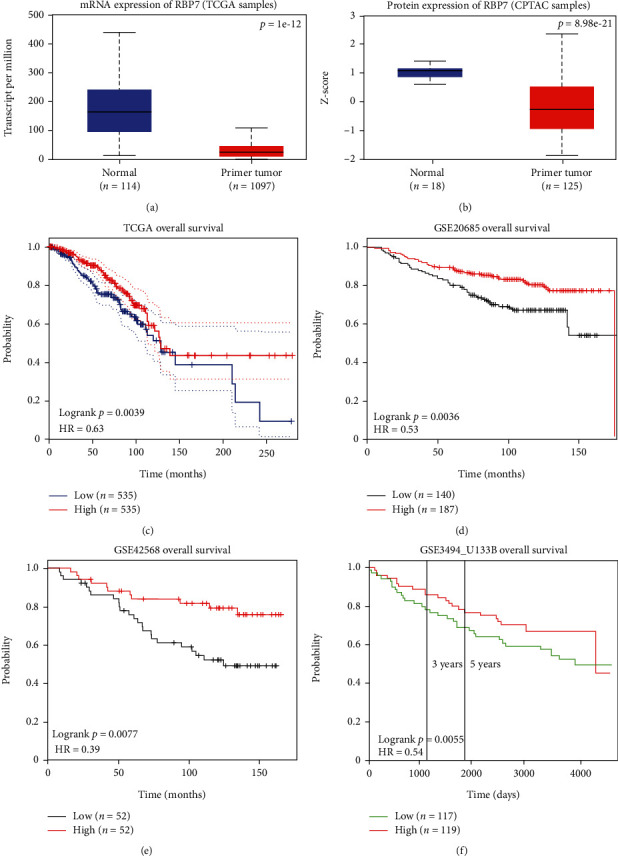
The mRNA and protein expression of *RBP7* in breast cancer and the overall survival analysis with *RBP7* mRNA expression. (a) Boxplot of the mRNA expression of *RBP7* in normal and breast cancer tissues. (b) Boxplot of the protein expression of *RBP7* in normal and breast cancer tissues. (c–f) Kaplan–Meier curve of OS based on the high and low expression of *RBP7* in breast cancer patients from different databases. A log-rank *p* value <0.05 was considered statistically significant.

To investigate the role of *RBP7* expression in breast cancer, the UALCAN database was utilized to analyze the expression of *RBP7* in 114 normal tissues and 1097 primary breast cancer tissues. The TCGA database results revealed that the mRNA expression level of *RBP7* was lower in breast cancer than in normal tissues (Figure 2(a)), which was further validated by the GSE37751 dataset from the GEO database (Supplementary Figure 1B). Then, we utilized the CPTAC database to analyze the protein expression of *RBP7* in breast cancer and found that the protein expression of *RBP7* in breast cancer was lower than that in normal tissues (Figure 2(b)), which was consistent with the result of *RBP7* expression in the TCGA database.

We subsequently utilized the transcriptomic sequencing data in the GEPIA database to assess the prognostic value of *RBP7* in breast cancer and found that a high level of expression of *RBP7* was favorable to the prognosis of breast cancer (Figure 2(c)). Kaplan–Meier survival analysis was performed with the GSE20685 (Figure 2(d)) and GSE42568 (Figure 2(e)) datasets from the GEO database to evaluate the prognostic value of *RBP7* in breast cancer, which showed that lower expression of *RBP7* was associated with a poorer prognosis in breast cancer. Furthermore, we used FROGgeneV2 to confirm the effect of *RBP7* on the OS of breast cancer patients. The results also showed that low expression of *RBP7* was significantly associated with a poor prognosis (Figure 2(f)). These results reveal that there is a significant association between *RBP7* expression and breast cancer prognosis and that *RBP7* serves as a protective factor in the prognosis of breast cancer.

### 3.3. *RBP7* Expression in Different Cells from Breast Cancer Tissues

**Figure 3:**
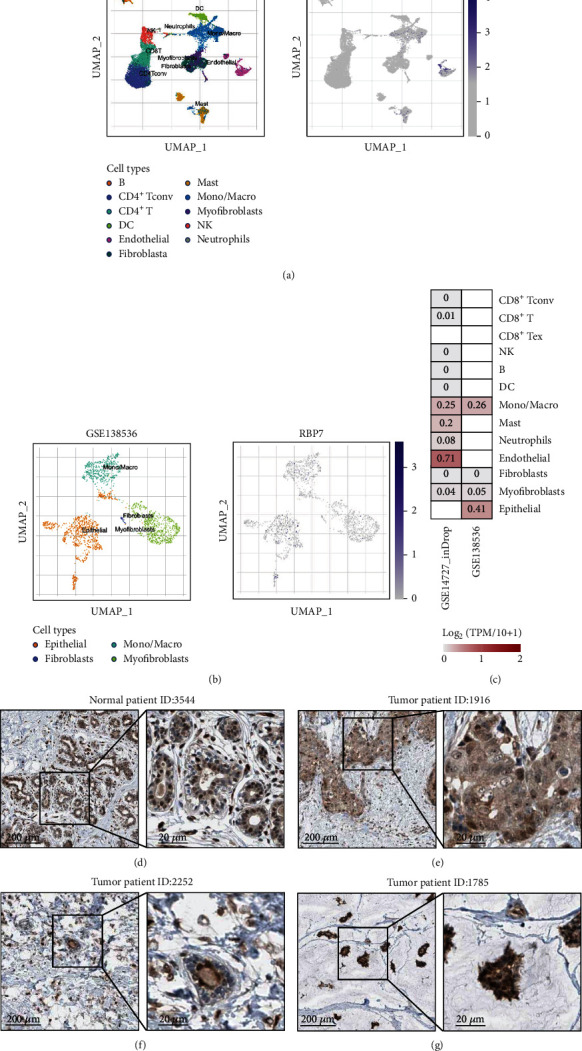
The cellular and subcellular localization of *RBP7* in breast cancer. (a–b) The cellular localization of *RBP7* mRNA in single breast cancer cells. The expression level is colored by marker intensity. UMAP: uniform manifold approximation and projection. (c) Heatmap showing the mRNA expression of *RBP7* in different cell types. (d–g) Representative immunohistochemical staining of normal tissues (https://www.proteinatlas.org/ENSG00000162444-RBP7/tissue/breast) (d) and breast cancer with weak (e), moderate (f), or strong (g) *RBP7* expression (https://www.proteinatlas.org/ENSG00000162444-RBP7/pathology/breast+cancer). Scale bars with lengths of 200 *μ*m or 20 *μ*m are displayed in the left and right panels, respectively.

We used the TISCH database to analyze the expression of *RBP7* in different cells from breast cancer tissues. It demonstrated that *RBP7* is mainly expressed in endothelial and epithelial cells in TISCH database (Figures 3(a) and 3(b)). A heat map of the gene module analysis depicts the expression of *RBP7* in different cell types of breast cancer datasets, in which endothelial and epithelial cells are characterized by high expression of *RBP7* (Figure 3(c)). Then, we utilized the immunohistochemistry (IHC) data detected by the HPA-034749 antibody from the HPA database to determine the protein expression of *RBP7* in breast cancer and normal tissues. The results showed that adipocytes were highly stained in normal breast tissues, while glandular and myoepithelial cells were mildly stained, mainly in the nucleus (Figure 3(d)). In the breast cancer samples, the expression level of *RBP7* in tumor cells was ranked as weak, moderate, and strong, which was scored by the staining intensity in the pathological IHC (Figures 3(e)–3(g)).Interestingly, as a nuclear receptor, *RBP7* was found to be mainly localized in the nucleus, indicating the important role of *RBP7* in the regulation of gene expression in epithelial cells of breast cancer tissues.

### 3.4. *RBP7* Coexpression Networks in Breast Cancer

**Figure 4:**
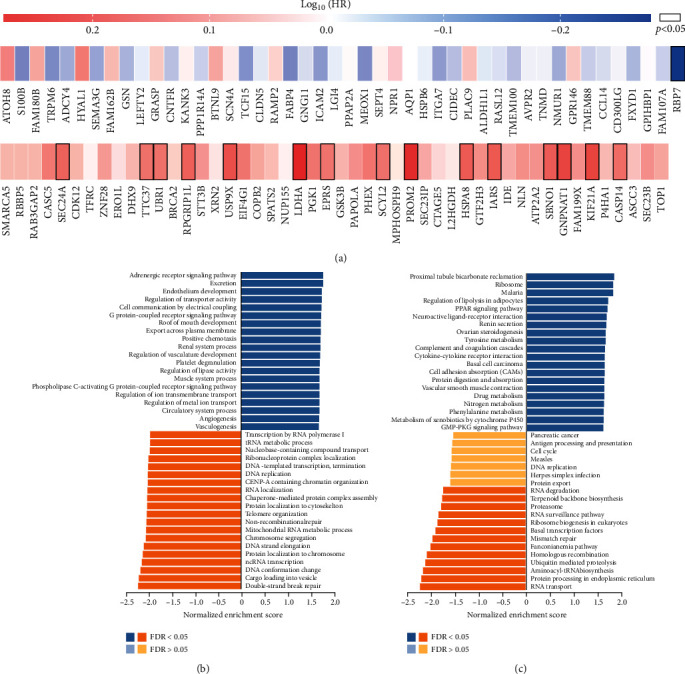
GO and KEGG pathway enrichment analysis of the coexpressed genes of *RBP7* in breast cancer. (a) Survival maps of the top 50 positive or negative coexpressed genes of *RBP7* in breast cancer. (b) GO_BP enrichment analysis of the coexpressed genes of *RBP7* in breast cancer. (c) KEGG pathway analysis of the coexpressed genes of *RBP7* in breast cancer.

To gain insight into the biological meaning of *RBP7* in breast cancer, the functional module of LinkedOmics was used to examine *RBP7* coexpression genes in the breast cancer cohort. The top 50 significant genes that were positively and negatively correlated with *RBP7* were selected as heat maps (Supplementary Figures 2A and 2B) in which *RBP7* displayed a strong positive association with the expression of FAM107A (*R* = 0.4767, *pvalue* =4.252e-01), GPIHBP1 (*R* = 0.4757, *pvalue* =8.560e-63), and FXYD1 (*R* = 0.4756, *pvalue* =9.258e-63).Remarkably, the top 50 negatively coexpressed genes had a high probability of being high-risk markers in breast cancer, of which 15 genes had significantly high HRs (*pvalue*<0.05). In contrast, there were no genes with high HRs (*p* < 0.05) in the top 50 positively coexpressed genes. These results further confirmed that *RBP7* performs a protective role in the progression of breast cancer (Figure 4(a)).

GO term annotation of biological processes showed that *RBP7* coexpressed genes mainly participate in the adrenergic signalling pathway, excretion, endothelium development, regulation of transporter activity, cell communication by electrical coupling and G protein-coupled receptor signalling pathway, with inhibition of the biological processes including double-strand break repair, cargo loading into vesicle, DNA conformation change, ncRNA transcription, and protein localization to chromosome (Figure 4(b)). KEGG pathway analysis showed that there was an enrichment in the regulation of lipolysis in adipocytes, PPAR signalling pathway, ovarian steroidogenesis, and drug metabolism (Figure 4(c)), indicating a widespread impact of *RBP7* on the global transcriptome.

### 3.5. *RBP7* Methylation in Breast Cancer

**Figure 5:**
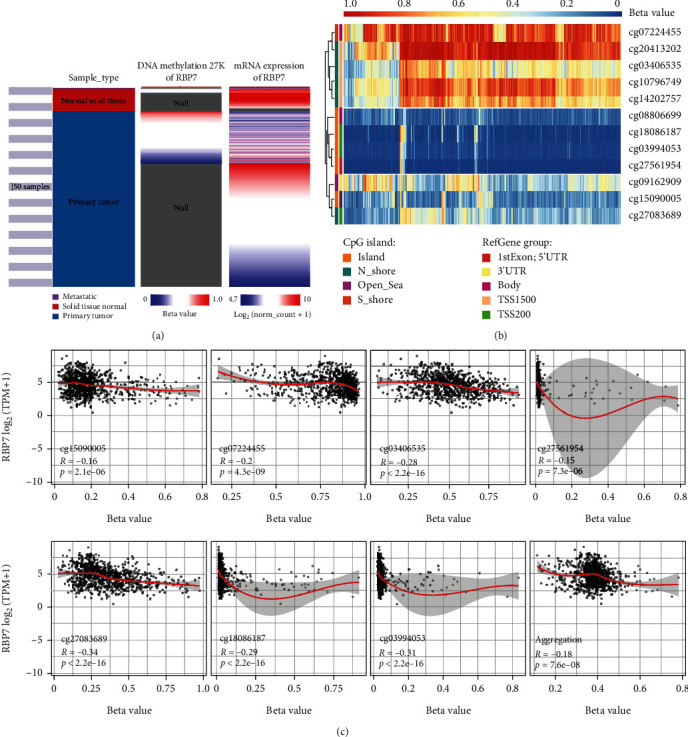
The methylation analysis of *RBP7* in breast cancer. (a) Heat maps for the mRNA expression and DNA methylation of *RBP7* in the TCGA database. (b) Visualization of methylation level and *RBP7* expression in different regions of the *RBP7* gene. (c) Analysis of the correlation between methylation on different methylation probes and mRNA expression of *RBP7* in breast cancer.

To further explore the mechanism of the differential expression of *RBP7* in breast cancer and normal tissues, we performed hierarchical clustering analysis of *RBP7* mRNA expression related to DNA methylation by using the UCSC Cancer Genomics Browser. The results showed that *RBP7* methylation mainly occurred in primary breast cancer, and the mRNA expression of RBP7 corresponding to hypermethylated samples is low (Figure 5(a)), indicating a potential correlation between mRNA expression and DNA methylation of *RBP7*.

Next, we performed methylation analysis with the Methsurv database and found that 4 methylation probes, namely, cg20413202, cg03406535, cg10796749, and cg14202757, in the promoter of *RBP7* were highly methylated in breast cancer (Figure 5(b)). As routinely done, the level of methylation was represented by a beta value: a beta value ≥0.6 was considered completely methylated, a beta value ≤0.2 was considered completely unmethylated, and a beta value between 0.2 and 0.6 was considered partially methylated. According to this standard, we found that the beta values of 3 of the 4 detected methylation probes, i.e., cg20413202 (beta value = 0.947), cg10796749 (beta value = 0.798), and cg14202757 (beta value = 0.694), were higher than 0.6, indicating that almost complete methylation occurred on the promoter of *RBP7* (Supplementary Figure 3A-D). Consistently, we found that most of the median beta values of different clinical stages were also above 0.6 (Supplementary Figure 3E-H), suggesting that promoter methylation leads to the inhibition of *RBP7* gene transcription in breast cancer.

Subsequently, we used the SMART App to verify the Spearman correlation between gene expression and DNA methylation of the probes of *RBP7* in breast cancer. The expression of *RBP7* was significantly negatively correlated with the methylation probes cg03406535, cg27083689, cg18086187, cg03994053, cg27561954, cg15090005, and cg07224455. The aggregation plot for all methylation probes showed that the expression level of *RBP7* was negatively correlated with the DNA methylation of *RBP7* (Figure 5(c)).

### 3.6. Clinicopathological Association of *RBP7* and Its Prognostic Value

**Figure 6:**
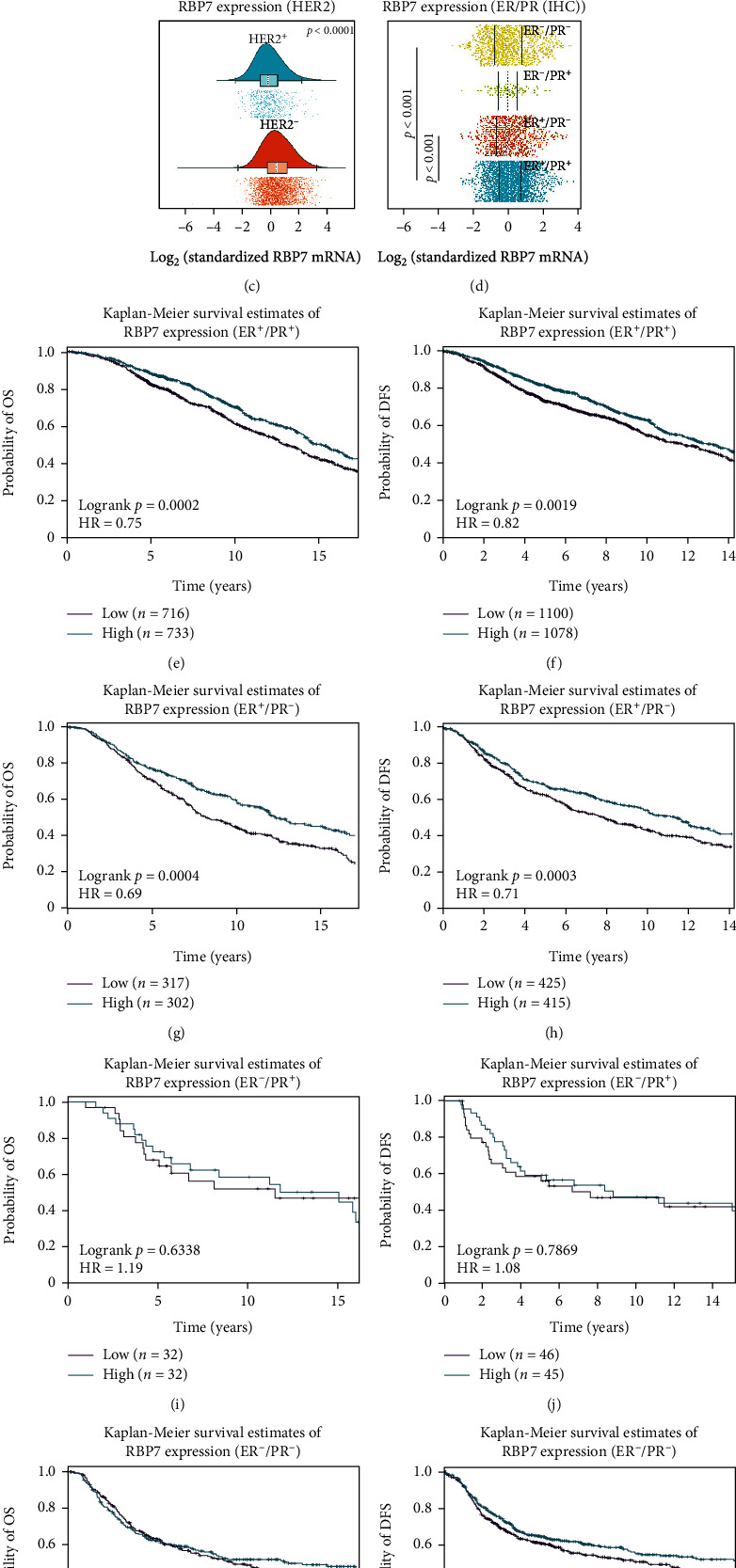
The mRNA expression of *RBP7* and the Kaplan–Meier survival curve in different molecular subtypes of breast cancer. (a–c) The mRNA expression of *RBP7* in different molecular subtypes, including ER (a), PR (b), and HER2 (c), of breast cancer. (d) Bee swarm plot of *RBP7* expression in breast cancer with positive or negative ER or PR expression. (e–l) Kaplan–Meier curves of breast cancer with the DNA microarray results of ER^+^/PR^+^ (e, f), ER^+^/PR^−^ (g, h), ER^−^/PR^+^ (i, j), or ER^−^/PR^−^ (k, l).

Breast cancer is a complex disease characterized by many morphological, clinical, and molecular features. Molecular subtypes and optimal treatments for breast cancer are usually based on immunohistochemical markers such as estrogen receptor (ER), progesterone receptor (PR), and human epidermal growth factor receptor 2 (HER2). We checked the relevance of *RBP7* expression and different clinicopathological features by using the web-based tool bc-GenExMiner. We found that the expression of *RBP7* was highly expressed in ER-positive breast cancer patients compared with ER-negative patients (Figure 6(a)). *RBP7* expression was higher in PR-positive breast cancer patients than in PR-negative patients (Figure 6(b)), whereas *RBP7* expression was higher in HER2-negative breast cancer patients than in HER2-positive patients (Figure 6(c)). To further determine the correlation of *RBP7* expression and hormone receptors (HRs), we utilized DNA microarray data to perform correlation analysis of *RBP7* expression with different combinations of ER and PR expression statuses, i.e., ER^+^/PR^+^, ER^+^/PR^−^, ER^−^/PR^+^, and ER^−^/PR^−^. The results showed that there were remarkable differences in *RBP7* mRNA expression in some hormone receptor combinations, i.e., ER^+^/PR^+^ vs. ER^−^/PR^−^, *p* < 0.01, and ER^+^/PR^+^ vs. ER^+^/PR^−^, *p* < 0.01 (Figure 6(d)).

Furthermore, we performed survival analysis of breast cancer patients with different ER/PR combinations. Downregulated *RBP7* expression was only significantly associated with poor prognosis in ER^+^/PR^+^ and ER^+^/PR^−^ patients but not in ER^−^/PR^+^ and ER^−^/PR^−^ breast cancer patients (Figures 6(e)–6(l)). OSbrca was used to verify the prognostic value of *RBP7* in these 4 groups with the TCGA database. Consistently, we found that lower *RBP7* expression was significantly correlated with poorer OS in ER^+^/PR^+^ and ER^+^/PR^−^ patients (Supplementary Figure 4).

### 3.7. Potential Regulatory Mechanisms and Target Drugs of *RBP7* in Breast Cancer

**Figure 7:**
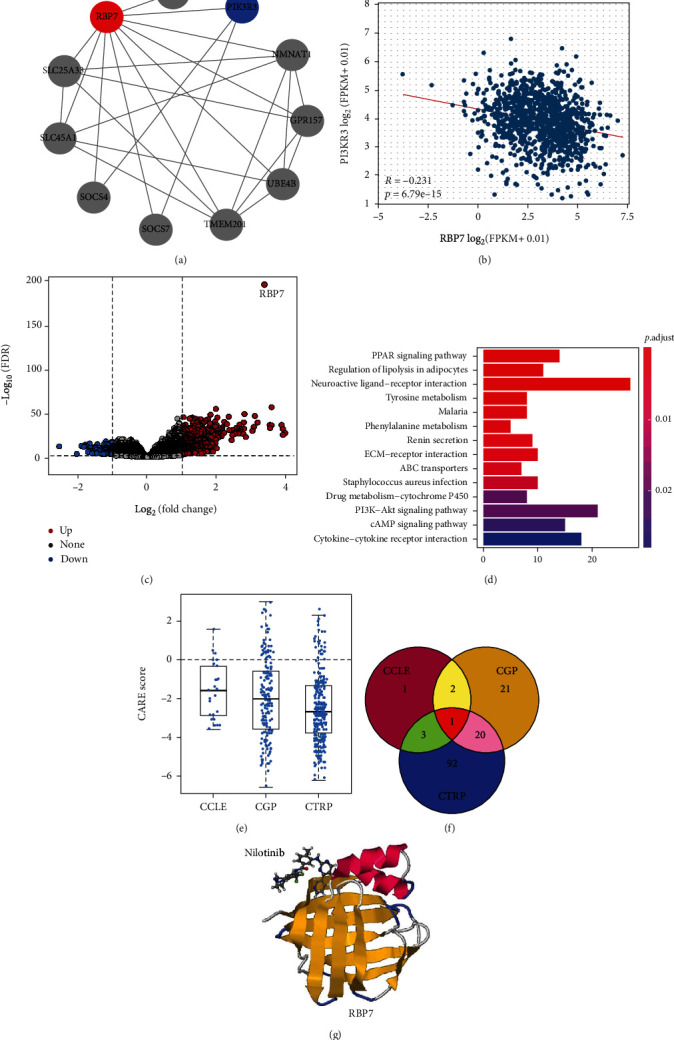
Analysis of the potential regulatory functions and *RBP7*-targeting drugs for breast cancer. (a) The PPI network for *RBP7* and its coexpressed genes by STRING. (b) Correlation analysis of *RBP7* and PIK3R3. (c) Volcano plot showing the differentially expressed genes in ER^+^ breast cancer with high or low expression of *RBP7*. (d) KEGG pathway analysis of DEGs in ER^+^ breast cancer with high or low expression of *RBP7*. (e) CARE analysis of the resistance module gene *RBP7* in the CCLE, CTRP and CGP databases. (f) Venn diagram showing the overlap of the *RBP7*-targeting drugs in the 3 databases. (g) Prediction of the binding sites between nilotinib and *RBP7*.

To further explore the PPI network of *RBP7* in breast cancer, we used STRING to identify genes that may interact with *RBP7*. The interactions between *RBP7* and proteins encoded by the functional genes, including SLC45A1, SLC25A33, UBE4B, TMEM201, NMNAT1, SOCS7, SOCS4, GPR157, PIK3R3, and POLR2G, are shown in Figure 7(a). Interestingly, in this interacting network, PIK3R3, a regulatory subunit of phosphatidylinositol 3-kinase (PI3K), was reported to play an important role in breast cancer. Therefore, we further analyzed the correlation between *RBP7* and PIK3R3 in breast cancer and found that the expression levels of *RBP7* and PIK3R3 were negatively correlated (*R* = −0.231, *p* = 6.79e − 15) (Figure 7(b)).

Then, we acquired ER^+^ breast cancer data from the TCGA database, which were quarterly ranked according to the expression level of *RBP7*. The high and low *RBP7* expression groups were defined as the first and fourth quarters of ER^+^ breast cancer data, respectively, and the differential genes (|log2(FC)| > 1) between these two groups were analyzed by using the Limma software package for display as a volcano plot (Figure 7(c)).

KEGG pathway analysis was conducted to investigate the functional implications of the DEGs, and it was found that several tumor-related pathways, such as the PPAR signalling pathway, regulation of lipolysis in adipocytes, and tyrosine metabolism, were significantly enriched (Figure 7(d)). In addition, the PI3K-AKT signalling pathway, which is important in ER^+^ breast cancers, was also enriched (Figure 7(d)).

Furthermore, we used the CARE database to analyze the association of the molecular alteration of *RBP7* with drug efficacy and found that *RBP7* was negatively correlated with drug efficacy in the CCLE, CGP, and CTRP databases (Figure 7(e)). Interestingly, we found that only one drug (nilotinib) was common among the resistant drugs from these 3 databases (Figure 7(f)). Finally, we visualized the binding site of *RBP7* with nilotinib by SwissDock (Figure 7(g)).

## 4. Discussion

*RBP7* is a member of the CRBP family and is involved in retinoic acid-mediated cellular responses [37]. Previous studies demonstrated that the retinol signalling pathway might be relevant to breast cancer progression. However, the prognostic value of *RBP7*, a new member of the CRBP family, in breast cancer is still unclear. In this study, we utilized various databases to explore the expression, prognosis, cellular localization, coexpression network, DNA methylation, and function of *RBP7* in breast cancer.

Gene expression analysis showed that *RBP7* is widely expressed in various normal tissues, including thyroid, testis, breast, and adipose tissues. Notably, in breast cancer tissues, *RBP7* is mainly expressed in epithelial cells with nuclear localization. Importantly, we found that both the mRNA and protein expression levels of *RBP7* in breast cancer tissues were significantly lower than those in normal tissues. Survival analysis with 4 different databases showed that low expression of *RBP7* was significantly associated with poor OS in breast cancer patients. To further illuminate the role of *RBP7* in the progression of breast cancer, we analyzed the expression and prognosis of *RBP7* in different molecular subtypes of breast cancer. The results showed that *RBP7* mRNA expression in ER-positive patients was higher than that in ER-negative patients, and higher expression of *RBP7* was associated with better OS and DFS in ER^+^ breast cancer patients, indicating that *RBP7* was mainly related to the prognosis of patients with ER^+^ breast cancer.

Our findings were consistent with other studies on the role of *RBP7* in breast cancer. For example, Kinyamu et al. used genome-wide transcriptional profiling to demonstrate that *RBP7* is positively regulated by estradiol (E2) in breast cancer cells [38]; Calvo et al. reported that blockers of estrogen receptors inhibited the expression of estradiol-modulated genes, including *RBP7*, in the mouse mammary gland [39]. It is reasonable to propose that upregulation of *RBP7* by E2 leads to a good prognosis in ER^+^ breast cancer. Previous studies demonstrated that the growth of human breast tumor cells is regulated by signalling pathways involving nuclear steroid thyroxine receptors [40], especially RARs, which show growth inhibitory activity against breast cancer cells both *in vitro* and *in vivo* [41].Interestingly, RAR and ER share a common coactivator, estradiol. Pemrick et al. proved that both RAR and ER have high affinity for *β*-estradiol by constructing chimeric RARs containing the ligand-binding domain of ER [42]. Furthermore, Fonja et al. found that anti-estrogen and Herceptin induced the expression of PDCD4, revealing that the intracellular crosstalk of RAR, ER, and Her-2 may play a role in growth inhibitory signalling in breast cancer cells [43]. In the retinoid pathway, RAR activation is associated with CRBP1-mediated retinol storage [9] [44]. *RBP7*, another member of the CRBP family, may also engage in crosstalk with RAR; thus, it may play an important role in mechanism regulation via RAR/ER in breast cancer. Based on the results above, we conclude that *RBP7* may be a tumor suppressor gene and that high expression of *RBP7* is associated with a good prognosis for ER^+^ breast cancer patients.

Our results demonstrated that the gene expression of *RBP7* in breast cancer was significantly lower than that in normal controls. Thus, we further explored the mechanism of *RBP7* downregulation in breast cancer. Bioinformatic analysis showed that there was a significant negative correlation between *RBP7* mRNA expression and promoter methylation. Breast cancer is a highly complex heterogeneous disease that forms different tumor subpopulations with distinct phenotypic characteristics. The different DNA methylation patterns between cell subpopulations drive the phenotypic changes in breast cancer, which is valuable for providing novel insights into intratumor epigenetic heterogeneity [45]. Recent studies have shown that methylation of the promoter as well as intragenic and intergenic regions is involved in the modulation of tumor development and invasion [46]. The methylation of CRBPs was reported to be associated with tumor development. For example, the methylation of CRBPs is common in the esophageal mucosa of patients with esophageal squamous cell carcinoma (ESCC) in the high-risk population and tends to increase in prevalence in foci with worse pathological changes [47]. The methylation profile of CRBPs in bladder cancers is also correlated with the clinicopathological features of poor prognosis [48]. DNA hypermethylation brings about epigenetic silencing of CRBPs in human and mouse breast cancer [49]. For example, CRBP1 gene silencing was found in 60% of G2 and 66.7% of G3 carcinoma cells due to CRBP1 promoter methylation [50]. DNA methylation can occur in the whole genome, including the promoter, gene body, 3′-untranslated region (UTR) and intergenic regions, while the promoter can be further divided into TSS200, TSS1500, 5′-UTR, and the 1st exon. DNA methylation in gene promoters generally has a negative regulatory effect on gene expression, whereas methylation in intragenic regions is not always associated with gene repression [51]. We utilized the MethSurv database to identify the methylation sites of *RBP7* in breast cancer and found 4 probes, namely, cg20413202, cg03406535, cg10796749, and cg14202757, located in TSS1500-N_Shore with high methylation. The analysis using SMART APP web tools showed that promoter methylation was negatively correlated with *RBP7* mRNA expression. In summary, our results demonstrate that the promoter methylation of *RBP7* be related to its transcriptional silencing, which may be a reasonable explanation for the gene downregulation of *RBP7* in breast cancer.

With the concept that low expression of *RBP7* leads to poor prognosis of ER^+^ breast cancer patients, we further performed KEGG pathway analysis in ER^+^ breast cancer and found that *RBP7* exerts its biological function through crosstalk with the PPAR and PI3K/AKT signalling pathways. PPARs represent a nuclear receptor superfamily that includes PPAR*α*, PPAR*β*/*δ*, and PPAR*γ*. It was reported that the activation of PPAR*γ* inhibits the cell growth of different cancers, such as colon cancer, gastric cancer, and liposarcoma [52]. For example, after cleavage by caspase-1 at Asp64, PPAR*γ* translocates to mitochondria, leading to attenuation of medium-chain acyl-CoA dehydrogenase (MCAD) activity and inhibition of fatty acid oxidation, which brings about the accumulation of lipid droplets and differentiation of tumor-associated macrophages (TAMs), thus resulting in an ultimate suppression of tumor growth [53, 54]. Another study by Mueller et al. reported that PPAR*γ* is highly expressed in human primary and metastatic breast cancer, and ligand activation of this receptor in breast cancer cells causes extensive lipid accumulation, which results in a reduction in the growth rate and clonogenic capacity of tumor cells [55] [56]. Intriguingly, application of the PPAR*γ* agonist rosiglitazone in combination with the MEK inhibitor trametinib can terminally differentiate breast cancer cells that have undergone epithelial-mesenchymal transition (EMT) into adipocytes [57]. As a PPAR*γ* target gene, *RBP7* is also an upstream regulator of some other PPAR*γ* target genes in the endothelium. PPAR*γ* and *RBP7* control the oxidative state of blood vessels by forming a transcriptional regulatory circuit (or hub) in the endothelium. Loss of *RBP7* impairs this regulatory circuit, resulting in oxidative stress and dysfunction in endothelial cells [58].Cancer cells have to endure oxidative stress throughout tumorigenesis, including during initiation, matrix detachment, transmission in the circulation, and relapse after therapy [59]. In addition, endothelial injury is closely related to tumorigenesis and accompanies malignant cancer cells in almost every stage of the metastatic process [60]. Thus, impairment of the regulatory circuit between *RBP7* and PPAR*γ* increases the opportunity to promote the occurrence of cancer.

**Figure 8:**
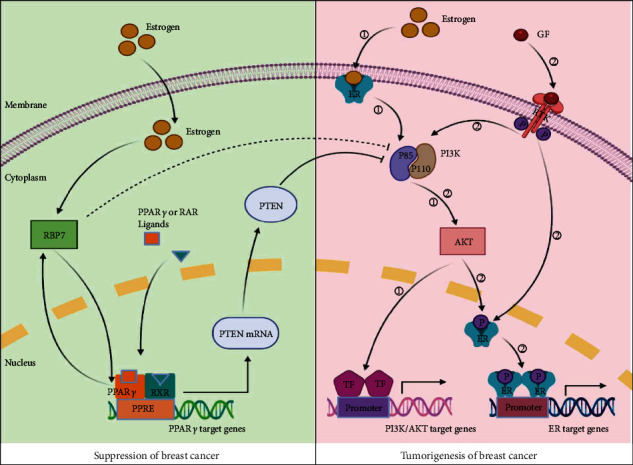
The hypothetical function of *RBP7* in the regulation of the PPAR and PI3K/AKT pathways in ER^+^ breast cancer.

PI3K/AKT is the most frequently activated signalling pathway that promotes tumor growth [61] and progression of breast cancer [62]. Bonofiglio *et al.* revealed that the ER*α* and PPAR*γ* pathways have an opposite effect on the regulation of the PI3K/AKT signal transduction cascade [63]. The nuclear receptor ER*α* has been shown to be involved in the pathophysiological process of breast cancer. Membrane-anchored ER*α* can activate various cytoplasmic kinases, including components of the PI3K/AKT pathway, through rapid nongenomic actions [64]. There are two signalling pathways involved in the activation of the PI3K/AKT pathway in ER^+^ breast cancer cells. The estrogen-dependent pathway activates the PI3K/AKT signalling process by directly binding to the p85*α* regulatory subunit of PI3K, thus enhancing the transcriptional activity of targeted genes in breast cancer cells [65]. For the estrogen-independent pathway, the interaction of EGFR with growth factor can directly induce ER*α* transcriptional activity through the PI3K/AKT signalling pathway [66]. Consequently, the downstream signalling of both pathways is activated, leading to the proliferation and survival of tumor cells. In contrast, PPAR*γ* can inhibit the PI3K/AKT pathway by upregulating PTEN transcription in breast cancer cells [63]. As an antagonist of the PI3K/AKT pathway, PTEN plays a key role in preventing tumorigenesis [67]. Based on our results and previous studies, we hypothesize that *RBP7* may regulate PTEN by targeting PPAR*γ*, thereby suppressing the activation of the PI3K/AKT pathway. The PPI network from bioinformatics analysis revealed that *RBP7* may directly interact with PIK3R3, resulting in activation of the PI3K/AKT pathway (Figure 8).

## 5. Conclusions

In conclusion, this study provides the first evidence that *RBP7* downregulation in breast cancer is associated with promoter methylation. Furthermore, we found that *RBP7* has prognostic value for ER^+^ breast cancer. Deep bioinformatics analysis of *RBP7*-related pathways reveals some vital information for the regulatory mechanism in ER^+^ breast cancer. However, these results need to be further validated by both in vitro and in vivo experiments. This study is helpful in providing novel approaches for clinical diagnosis and treatment.

## Data Availability

The original contributions presented in the study are included in the article/Supplementary material, and further inquiries can be directed to the corresponding authors.

## Supplementary Materials

Supplementary Materials Supplementary Figure 1. Analysis of the mRNA expression of RBP7 in breast cancer. Supplementary Figure 2. The genes correlated with the expression of RBP7. Supplementary Figure 3. Density maps of CpG island methylation and the methylation distribution in different clinical stages of breast cancer. Supplementary Figure 4. Effect of RBP7 expression on the overall survival curves of breast cancer patients of different molecular subtypes.
