# Supplementary material for: Methylation-Mediated Silencing of RBP7 Promotes Breast Cancer Progression through PPAR and PI3K/AKT Pathway
Source: J Oncol. 2022 Oct 13;2022:9039110. doi: 10.1155/2022/9039110 (PMC9584705; doi:10.1155/2022/9039110)
Supplement: Supplementary Materials — Supplementary Figure 1. Analysis of the mRNA expression of RBP7 in breast cancer. Supplementary Figure 2. The genes correlated with the expression of RBP7. Supplementary Figure 3. Density maps of CpG island methylation and the methylation distribution in different clinical stages of breast cancer. Supplementary Figure 4. Effect of RBP7 expression on the overall survival curves of breast cancer patients of different molecular subtypes. [file 9039110.f1.zip › Supplementary Figure 3.docx]

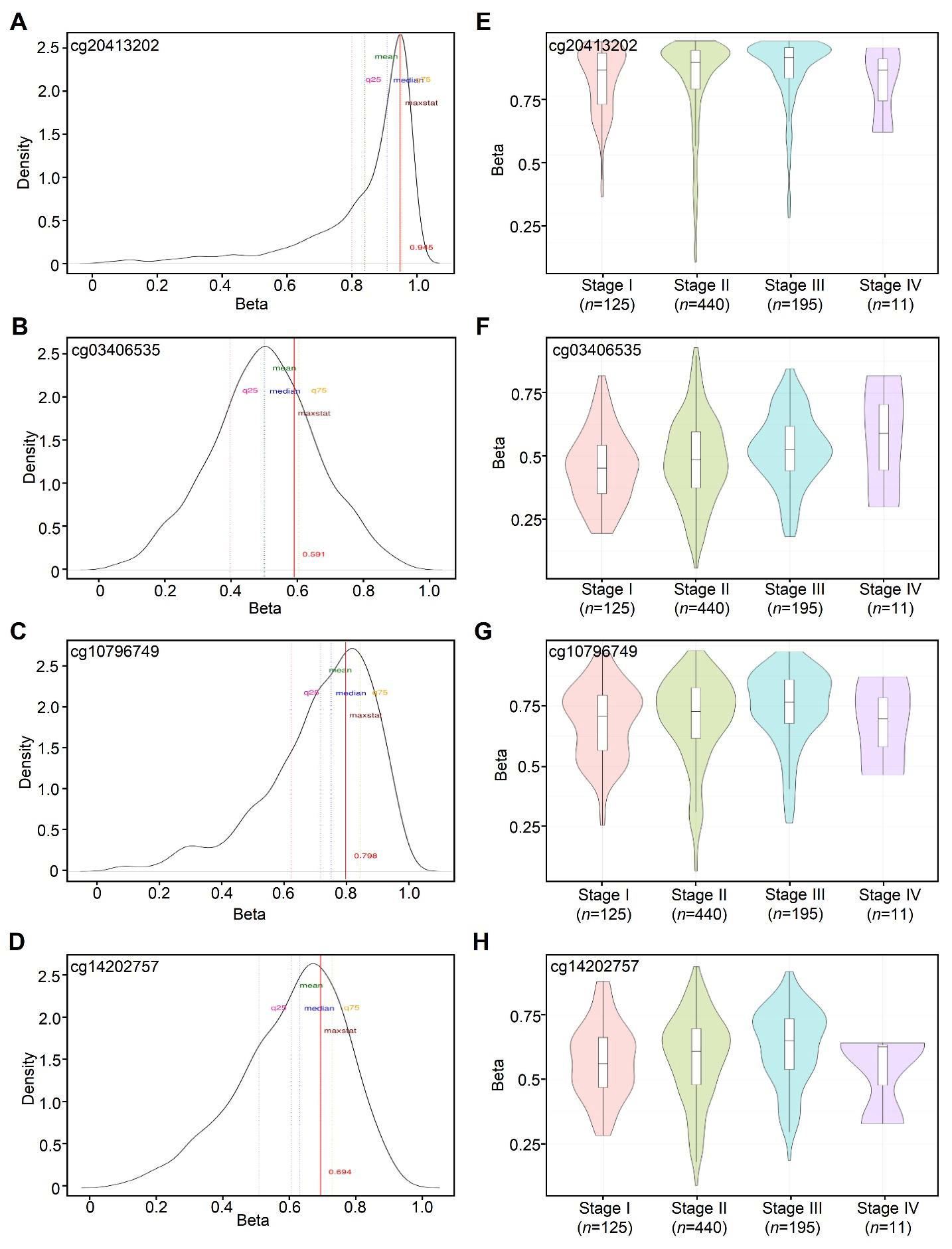


**Supplementary Figure 3. Density maps of CpG island methylation and the methylation distribution in different clinical stages of breast cancer.**

(**A-D**) Density map of the methylation of TSS1500-N_Shore probes, including cg20413202 (A), cg03406535 (B), cg10796749 (C) and cg14202757 (D).

(**E-H**) Methylation profiles of TSS1500-N_Shore probes, including cg20413202 (E), cg03406535 (F), cg10796749 (G) and cg14202757 (H), for different clinical stages of breast cancer.
